# A Case of Hypersexuality in a Patient Receiving Aripiprazole for Schizophrenia

**DOI:** 10.1155/2021/5557211

**Published:** 2021-06-24

**Authors:** Lakshmi Priya, Bini Moorthy

**Affiliations:** Department of Psychiatry, University of Missouri-Kansas City School of Medicine, 2411 Holmes Street Kansas City, MO 64108, USA

## Abstract

Aripiprazole is an antipsychotic with partial agonist activity at the dopamine D2 receptor which may lead to compulsive behaviors including hypersexuality. The case presented is of a 24-year-old male with schizophrenia who developed hypersexuality symptoms after initiation of aripiprazole. Cessation of the aripiprazole led to a reduction in hypersexual behaviors. Clinicians should be aware of compulsive behaviors including hypersexuality with aripiprazole use and consider discontinuation of aripiprazole treatment in patients suffering from hypersexuality.

## 1. Introduction

Aripiprazole is a second-generation antipsychotic commonly used to treat schizophrenia and bipolar disorder. It has unique properties compared to other antipsychotics as it is a partial agonist at the dopamine D2 receptor [1]. It is also a partial agonist at the 5-HT1A receptor and antagonist at the 5-HT2A receptor [1]. Aripiprazole is often favored compared to other antipsychotics due to its lower side effect profile. Common side effects include akathisia, secondary Parkinsonism, and acute dystonia [1]. However, it is associated with lower extrapyramidal and metabolic symptoms compared to other antipsychotics [1]. Many antipsychotics also have sexual side effects as they block dopamine receptors which increase prolactin levels causing decreases in libido [2]. Aripiprazole, however, has an agonist activity at the dopamine receptor which can induce compulsive behaviors such as gambling, shopping, and hypersexuality [2]. There are case reports in the literature of hypersexuality following aripiprazole treatment [2–7]. In these cases, hypersexuality symptoms arose days to weeks after administration of the medication. In addition, discontinuation of aripiprazole decreased hypersexuality symptoms within a few weeks to a month. In 2016, the United States Food and Drug Administration (FDA) released a warning statement regarding increased impulse control problems in patients receiving aripiprazole including compulsive eating, shopping, and sexual actions [8]. Patients at a higher risk of developing impulsive behaviors include those with a personal or family history of obsessive-compulsive disorder, impulse control disorder, bipolar disorder, impulsive personality, alcoholism, drug abuse, or other addictive behaviors [8]. Here, we present a case of a 24-year-old male who developed hypersexuality symptoms after receiving aripiprazole as treatment for schizophrenia.

## 2. Case Presentation

The patient was a 24-year-old male with a past psychiatric history of schizophrenia who was seen in the outpatient clinic due to hypersexuality after starting aripiprazole. The patient had no known health problems. He had no history of head trauma, seizures, sexually transmitted disease, or significant substance abuse problems. He was first diagnosed with schizophrenia at the age of 19 years and was started on olanzapine, which he responded well to for years. The patient was living with his mother and had completed 11^th^ grade level education. He did not have any history of developmental or intellectual difficulties. He was working part time in a fast food restaurant when he started to have the decline. He was single, never married, and had no significant other. The patient did not have very many acquaintances and led a quiet life. He has no known legal issues so far. The patient used cannabis occasionally when he was 17, but denied any use now. He did not have any problems with alcohol and or other illicit substances. He did not have any history of tobacco use. There was no known family history of mental illness.

The patient was previously stable on olanzapine 20 mg/day for four years and then became noncompliant due to unclear reasons, leading to worsening of psychotic symptoms. He was switched to oral aripiprazole 15 mg/day with a plan to switch to a long-acting injectable due to possible nonadherence. During the initial oral one-week trial, the patient became increasingly unstable, with physical and verbal altercations with his mother. The patient also ran away from home and lost his job due to public masturbation at his workplace. His mother also noticed that the patient was increasingly flirtatious and inappropriate in public with females, which were behaviors he had never displayed previously. Given his worsening behaviors, the patient was admitted to the inpatient unit and was switched to intramuscular injection (IM) of aripiprazole 400 mg, assuming that he would benefit from a long-acting injectable and that he could be non-adherent with the oral medication. He became increasingly sexually aroused and was acting inappropriate in the days following. During the hospital stay, there was an instance where he had sex with a female peer and had to be placed on 1 : 1 observation, i.e., one staff was assigned to continuously observe the patient. After discharge from the hospital, the patient needed constant reminders in his group home to keep his hands out of his pants. Despite these attempts to reduce the inappropriate behavior, the patient endorsed that his sexual arousal was more than normal after starting the aripiprazole. At this point in time, the patient had received two doses of aripiprazole 400 mg IM. While on aripiprazole, he did not show any gambling urges and or other impulsivity other than the sexual preoccupations. The sexual preoccupations started within a week of starting with aripiprazole. Further aripiprazole injections were discontinued, and the patient was again prescribed olanzapine 20 mg/day given his previous good response. In the group home, the patient continued to remain occasionally agitated with outbursts and required constant redirection about appropriate sexual behaviors in public. He also continued to display sexually inappropriate behaviors towards females in a day program and was banned from attending the program until he was able to behave appropriately. Although he had reduction in the intense sexual behaviors four weeks after stopping the aripiprazole injections, he did not completely return to baseline. Given his ongoing sexually inappropriateness and possible reports of being internally stimulated, he was started on paliperidone 6 mg/day for augmentation and with plan to taper and stop olanzapine. The patient started to have fewer outbursts and was noted to be in much better mood after three to four weeks of starting the paliperidone. He was noted to be less sexually preoccupied and with reduction in inappropriate behaviors in public. When writing this report, the patient was on both olanzapine 20 mg/day and paliperidone 6 mg/day, with the goal of attaining paliperidone monotherapy, initially oral and subsequently long-acting injections. The patient continued to live in the group home to learn social skills before moving into independent housing. After 12 weeks of paliperidone use, the patient was completely free from hypersexual thoughts and behaviors.

## 3. Discussion

This case highlights the rare hypersexuality side effect that can arise in patients receiving aripiprazole for schizophrenia treatment. This is likely due to aripiprazole's partial agonistic effect on the dopamine D2 receptor [1]. Clinicians should be aware of the increased risk of hypersexuality and other impulsive behaviors as they can significantly impair a patient's daily functioning.

Olanzapine has also been reported to cause hypersexuality in individuals and with return to baseline once the medication has been discontinued [7]. It is interesting to note that the patient presented here took olanzapine previously for four years without any complaints of hypersexuality. The primary psychotic symptoms of auditory hallucinations and paranoia were clearly controlled previously on olanzapine without any side effects of akathisia, extrapyramidal symptoms, or hypersexuality.

The hypersexual behaviors were emergent in this case after the initiation of the oral aripiprazole dose and then intensified after being switched to the injectable long-acting medication. The half-life of oral aripiprazole is approximately 75 hours compared to the half-life of a 400 mg long-acting injectable, which is 46.5 days [1, 9]. It took a considerable time, approximately 12 weeks before the hypersexuality symptoms finally disappeared, which may be explained by the long half-life of the aripiprazole injectable. It is also unclear whether switching to olanzapine caused a prolongation in hypersexual behaviors, compared to other case reports of a quicker return to normal following discontinuation of aripiprazole [3, 4].

Psychosocial risk factors and vulnerabilities of this patient as a young adult getting the opportunities to interact with female counterparts in a setting outside of his controlled home environment could have also led to prolongation of symptoms despite discontinuation of aripiprazole. Being an early young adult could have hindered the patient from endorsing hypersexuality as a side effect, and his age may have led him to engage in behaviors with little insight. Parents raising young adults may find such behaviors embarrassing to report leading to delays in reporting until a social or public issue arises.

Screening patients for these impulsive symptoms at regular visits may decrease the risk of impairing interpersonal relationships and prevent problems with the law. In addition, clinicians could consider taking precautions when prescribing antipsychotics to patients who have psychiatric comorbidities that place them at a higher risk of impulsive or addictive behaviors. Dose reduction or complete cessation of treatment should be considered as previous cases have shown resolution of hypersexuality symptoms following these measures. Clinicians should also consider the age of the patient and psychosocial vulnerabilities that could lead to prolongation of symptoms despite medication discontinuation.

## Data Availability

All data is included in the case report.
